# Raw data of silver extraction from sodium-silver jarosite using three different lixiviants in alkaline medium

**DOI:** 10.1016/j.dib.2021.107511

**Published:** 2021-10-26

**Authors:** Hernán Islas, Mizraim U. Flores, Julio C. Juárez, Martín Reyes, Alien Blanco, Emmanuel J. Gutiérrez, Javier Aguilar, Mary C. Nolasco, Israel Rodríguez, Iván A. Reyes

**Affiliations:** a Escuela Superior de Zimapán, Universidad Autónoma del Estado de Hidalgo, Zimapán, Hidalgo 42337, México; b Área de Electromecánica Industrial, Universidad Tecnológica de Tulancingo, Tulancingo, Hidalgo 43642, México; c Área Académica de Ciencias de la Tierra y Materiales, Universidad Autónoma del Estado de Hidalgo, Mineral de la Reforma, Hidalgo 42183, México; d División de Mecánica, Tecnológico de Estudios Superiores de Tianguistenco, Santiago Tianguistenco, Estado de México 52650, México; e Instituto de Metalurgia, Universidad Autónoma de San Luis Potosí, San Luis Potosí, San Luis Potosí 78210, México; f Consejo Nacional de Ciencia y Tecnología, Benito Juárez, Ciudad de México 03940, México

**Keywords:** Hydrometallurgy, Silver leaching, Jarosite-type compounds, Complexing agent, Batch experiments

## Abstract

This article presents the raw data of silver concentration ([Ag]) obtained as a function of time (t) from silver leaching experiments, which were conducted using a synthetic sodium-silver jarosite and different complexing agents: thiosulfate, thiocyanate, and cyanide. Leaching experiments were performed under different conditions of temperature, pH and lixiviant concentration. The data refer to the article “Silver leaching from jarosite-type compounds using cyanide and non-cyanide lixiviants: a kinetic approach” (Islas et al., 2021), in which they were used to determine the leaching kinetics of jarosite-type compounds. The datasets were obtained experimentally from batch experiments. Concentration of silver, [Ag], was determined in each experiment as a function of time by atomic absorption spectroscopy. The information presented in this article can be useful for engineering students interested in mineral processing; particularly, for the calculation of kinetic parameters of silver leaching process. The data could also help in the formulation, implementation, or optimization of strategies for extraction of valuable metals from residues generated by the hydrometallurgical industry.

## Specifications Table

**Table:** 

Subject	Engineering
Specific subject area	Mineral Processing; Hydrometallurgy
Type of data	Table
How the data were acquired	[Ag] in leaching samples was determined by atomic absorption spectroscopy (AAS), in a PerkinElmer Spectrometer model AAnalyst 200 AA (Waltham, MA, USA).
Data format	Raw
Description of data collection	Silver leaching from jarosite samples was evaluated by monitoring the variation of silver extraction with temperature (T), concentration and type of complex (S_2_O_3_^2−^, SCN^−^ or CN^−^), and initial pH. For each experiment, one parameter was modified, while the other three were kept constant. To follow the progress of the leaching reaction in each experiment, the concentration of silver, [Ag], was determined by AAS in samples of 5 ml, which were taken from the leaching solution at different times (t).
Data source location	Institution: Autonomous University of San Luis Potosi City/Town/Region: San Luis Potosí, S.L.P. Country: Mexico
Data accessibility	The datasets are deposited on Mendeley Data and can be accessed via the following link: https://doi.org/10.17632/wj8w88
Related research article	H. Islas, M.U. Flores, J.C. Juárez, M. Reyes, A. Blanco, E.J. Gutiérrez, J. Aguilar, M.C. Nolasco, I. Rodríguez, I.A. Reyes, Silver leaching from jarosite-type compounds using cyanide and non-cyanide lixiviants: a kinetic approach, Miner. Eng. 174 (2021) 107250. 10.1016/j.mineng.2021.107250

## Value of the Data

• The datasets presented can be useful for scientists to develop comparative studies of silver leaching from ores using alternatives to cyanide such as thiosulfate and thiocyanate, aiming to increase the silver extraction and to minimize the reagent consumption.
• The datasets can be useful for engineering students as case study of leaching process to calculate reaction rates and kinetic parameters.
• The datasets can help scientists and engineers in the formulation, implementation, or optimization of strategies to recover valuable metals from residues produced by the hydrometallurgical industry.

## 1 Data Description

The datasets presented in this article show the results (silver concentration vs. time) regarding the leaching of a synthetic sodium-silver jarosite sample with three different lixiviants: thiosulfate, thiocyanate, and cyanide. Table 1 shows the results of the silver leaching from the sodium jarosite sample with thiosulfate. It shows the effects of temperature, pH, and concentration of complexing agent. The range in which these variables were evaluated during the experiments is also shown in Table 1. The results of [Ag] against time for the silver leaching with thiocyanate are shown in Table 2. Table 3 shows the results of [Ag] vs. time for silver leaching with cyanide.

**Table 1:** Datasets of [Ag] vs. time for silver leaching evaluation in alkaline medium from sodium-silver jarosite using S_2_O_3_^2−^ as complexing agent. [Ag] in mg L^−1^ and t in min.

NaS_2_O_3_ (mol L^−1^)/pH_i_^a^																				
0.1	t	0	2	6	10	15	20	25	30	40	60	80	120	-	-	-	-	-	-	-
11.84	[Ag]	0	0.045	0.32	1.382	2.268	2.955	3.375	3.631	3.762	3.754	3.608	3.684	-	-	-	-	-	-	-

0.04081	t	0	1	2	4	6	8	10	15	20	25	30	40	60	90	-	-	-	-	-
11.80	[Ag]	0	0	0	0.001	0.094	0.304	1.03	3.079	4.639	5.636	6.338	6.788	6.777	6.671	-	-	-	-	-

0.02857	t	0	2	6	10	15	20	30	40	60	80	110	150	-	-	-	-	-	-	-
11.80	[Ag]	0	0.103	0.162	0.351	0.898	2.079	3.953	4.924	5.598	5.706	5.708	5.589	-	-	-	-	-	-	-

0.01224	t	0	1	2	4	6	8	10	15	20	25	30	35	40	50	60	80	120	-	-
11.77	[Ag]	0	0.004	0	0.001	0.001	0.001	0.001	0.01	0.122	0.744	1.098	1.414	1.628	2.014	2.252	2.366	2.386	-	-

0.00408	t	0	2	6	10	15	20	30	40	60	90	120	150	225	-	-	-	-	-	-
11.85	[Ag]	0	0.125	0.184	0.23	0.405	0.445	1.018	2.113	3.983	4.844	4.962	5.008	5.016	-	-	-	-	-	-

0.00245	t	0	2	6	10	15	20	30	40	60	80	120	180	-	-	-	-	-	-	-
11.88	[Ag]	0	0	0.035	0.044	0.062	0.123	0.265	0.81	2.088	2.981	3.854	4.083	-	-	-	-	-	-	-

0.000408	t	0	2	6	10	15	20	30	40	60	80	120	180	240	300	-	-	-	-	-
11.89	[Ag]	0	0	0	0	0	0	0.066	0.26	0.974	1.638	2.576	2.897	2.955	2.985	-	-	-	-	-

NaOH (mol L^−1^) /pH_i_																				
0.06	t	0	0.5	1	1.5	2	2.5	3	4	5	6	8	10	12	15	20	25	30	40	60
12.73	[Ag]	0	0.11	0.132	0.17	0.5	1.073	1.852	2.994	4.027	4.968	6.526	7.754	8.274	8.687	8.519	8.594	8.669	8.813	8.658

0.03	t	0	1	2	3	4	5	6	8	10	12	14	16	18	20	25	30	40	60	-
12.39	[Ag]	0	0.069	0.131	0.256	0.356	0.706	1.285	2.575	3.544	4.402	4.952	5.359	5.664	5.778	5.689	5.7	5.319	5.213	-

0.01	t	0	1	2	4	6	8	10	15	20	25	30	40	60	90	-	-	-	-	-
12.03	[Ag]	0	0	0	0.001	0.094	0.304	0.871	2.779	4.139	5.436	6.178	6.658	6.777	6.671	-	-	-	-	-
0.0066	t	0	2	6	8	10	15	20	25	30	40	50	60	90	120	-	-	-	-	-
11.80	[Ag]	0	0	0.016	0.095	0.225	1.109	2.387	3.548	4.417	5.782	6.431	6.655	6.571	6.437	-	-	-	-	-

0.0011	t	0	2	4	6	10	15	20	25	30	40	50	60	80	100	120	150	240	-	-
11.08	[Ag]	0	0	0	0.002	0.013	0.042	0.101	0.23	0.593	1.112	2.471	3.565	5.067	6.016	6.531	6.707	6.345	-	-

0.00055	t	0	5	10	15	20	30	40	50	60	80	100	120	150	180	220	300	-	-	-
10.32	[Ag]	0	0.023	0.034	0.06	0.081	0.189	0.309	0.445	0.577	1.773	2.971	4.143	5.379	6.206	6.899	6.942	-	-	-

0.000275	t	0	5	10	15	20	30	40	60	80	100	120	150	180	220	300	380	480	-	-
9.98	[Ag]	0	0.005	0	0.003	0.01	0.013	0.009	0.179	0.482	1.171	1.968	2.958	3.891	5.068	6.216	6.452	6.632	-	-

T (°C) /pH_i_																				
70	t	0	0.25	0.5	0.75	1	1.25	1.5	1.75	2	2.5	3	3.5	4	5	6	8	10	-	-
10.59	[Ag]	0	0.024	0.064	0.307	0.571	1.045	1.366	1.978	2.354	3.215	3.773	4.381	5.128	6.02	6.837	7.333	7.414	-	-

60	t	0	0.25	0.5	0.75	1	1.25	1.5	1.75	2	3	3.5	4	5	6	8	10	12	15	20
10.92	[Ag]	0	0.002	0.024	0.113	0.222	0.49	0.656	1.066	1.522	2.663	3.308	4.109	5.024	6.13	7.683	8.645	9.307	9.239	9.358

50	t	0	0.25	0.5	0.75	1	1.25	1.5	2	2.5	3	4	6	8	12	15	20	25	-	-
11.31	[Ag]	0	0.001	0.015	0.025	0.045	0.096	0.144	0.213	0.499	0.704	1.56	3.216	4.246	5.446	5.99	6.348	6.41	-	-

45	t	0	0.5	1	1.5	2	3	4	6	8	10	15	20	25	30	-	-	-	-	-
11.57	[Ag]	0	0.003	0	0.054	0.08	0.32	0.993	2.195	3.364	4.272	5.509	6.164	6.309	6.45	-	-	-	-	-

40	t	0	0.5	1	1.5	2	3	4	6	8	10	15	20	25	30	40	50	60	-	-
11.83	[Ag]	0	0.002	0.001	0.052	0.051	0.193	0.416	1.609	2.723	3.779	5.313	6.357	6.793	6.98	7.053	7.025	6.635	-	-

35	t	0	1	2	4	6	8	10	15	20	25	30	40	50	60	80	-	-	-	-
12.04	[Ag]	0	0.002	0.028	0.089	0.31	0.732	1.621	3.321	4.476	5.069	5.49	5.653	5.759	5.518	5.748	-	-	-	-

30	t	0	1	2	4	6	8	10	15	20	25	30	40	60	90	-	-	-	-	-
12.33	[Ag]	0	0	0	0.001	0.094	0.304	0.871	2.779	4.139	5.436	6.178	6.658	6.777	6.671	-	-	-	-	-


a pH_i_ = initial pH, kept constant in all experiments.

**Table 2:** Datasets of [Ag] vs. time for silver leaching evaluation in alkaline medium from sodium-silver jarosite using SCN^−^ as complexing agent. [Ag] in mg L^−1^ and t in min.

NaSCN (mol L^−1^)/pH_i_																				
0.1	t	0	2	4	6	10	15	20	25	30	40	50	60	90	-	-	-	-	-	-
11.77	[Ag]	0	0.011	0.173	0.478	1.008	1.622	2.208	2.623	2.914	3.188	3.194	3.277	3.337	-	-	-	-	-	-

0.04081	t	0	1	2	4	6	8	10	15	20	25	30	40	50	60	80	100	-	-	-
11.95	[Ag]	0	0.01	0.012	0.013	0.041	0.095	0.173	0.379	0.537	0.653	0.762	0.91	0.993	0.963	0.883	0.916	-	-	-

0.02857	t	0	2	4	6	10	15	20	25	30	40	50	60	80	120	-	-	-	-	-
11.82	[Ag]	0	0.008	0.011	0.019	0.043	0.162	0.286	0.396	0.498	0.659	0.768	0.854	0.917	0.945	-	-	-	-	-

0.01224	t	0	1	2	3	4	6	8	10	15	20	25	30	40	50	60	80	100	120	-
11.83	[Ag]	0	0	0	0	0	0	0.002	0.007	0.023	0.042	0.068	0.087	0.119	0.145	0.171	0.206	0.226	0.237	-

0.00408	t	0	3	6	10	15	20	30	40	60	80	120	180	-	-	-	-	-	-	-
11.85	[Ag]	0	0.001	0.004	0.006	0.016	0.028	0.058	0.086	0.141	0.182	0.211	0.218	-	-	-	-	-	-	-

0.00245	t	0	2	4	6	10	15	20	30	40	60	80	120	180	-	-	-	-	-	-
11.86	[Ag]	0	-0.013	-0.029	-0.029	-0.025	-0.033	0.002	-0.009	-0.007	-0.011	0.019	0.03	0.04	-	-	-	-	-	-

0.000408	t	0	3	6	10	15	20	30	40	60	80	120	180	240	-	-	-	-	-	-
11.86	[Ag]	0	0.019	-0.003	-0.021	-0.032	-0.031	-0.048	-0.051	-0.041	0.015	0.045	0.056	0.051	-	-	-	-	-	-

NaOH (mol L^−1^) /pH_i_																				
0.06	t	0	0.5	1	1.5	2	3	4	6	8	10	12	15	20	40	-	-	-	-	-
12.56	[Ag]	0	0.005	0.019	0.028	0.037	0.098	0.228	0.497	0.696	0.839	0.928	1.018	0.978	1.118	-	-	-	-	-

0.03	t	0	1	2	3	4	6	8	10	12	16	20	25	30	40	50	-	-	-	-
12.33	[Ag]	0	0.002	0.016	0.024	0.032	0.114	0.232	0.35	0.478	0.748	0.902	1.057	1.157	1.118	1.176	-	-	-	-

0.01	t	0	1	2	4	6	8	10	15	20	25	30	40	50	60	80	100	-	-	-
11.95	[Ag]	0	0.01	0.012	0.013	0.015	0.055	0.193	0.329	0.537	0.683	0.762	0.88	0.993	0.963	0.883	0.916	-	-	-

0.0066	t	0	1	2	4	6	10	15	20	25	30	40	50	60	80	120	180	240	-	-
11.64	[Ag]	0	0.002	0.004	0.005	0.006	0.008	0.043	0.222	0.341	0.494	0.742	0.937	1.131	1.351	1.478	1.521	1.429	-	-
0.00275	t	0	2	4	8	12	16	20	25	30	40	50	60	80	100	120	150	180	220	280
11.34	[Ag]	0	0.002	0.004	0.006	0.008	0.011	0.016	0.093	0.153	0.346	0.596	0.726	0.905	1.026	1.112	1.179	1.199	1.219	1.242

0.0011	t	0	2	4	6	10	15	20	30	40	60	80	120	180	240	280	420	-	-	-
10.88	[Ag]	0	0.003	0.008	0.011	0.017	0.022	0.029	0.139	0.234	0.463	0.787	1.119	1.477	1.581	1.603	1.577	-	-	-

0.00055	t	0	4	10	15	20	25	30	40	50	60	80	110	140	180	220	340	400	-	-
10.38	[Ag]	0	0	0.001	0.002	0.004	0.006	0.009	0.018	0.027	0.114	0.23	0.503	0.754	0.994	1.117	1.358	1.34	-	-

T (°C) /pH_i_																				
70	t	0	0.25	0.5	0.75	1	1.25	1.5	2	2.5	3	3.5	4	5	6	8	10	15	20	-
10.59	[Ag]	0	0.043	0.088	0.174	0.224	0.31	0.416	0.586	0.74	0.864	0.976	1.067	1.286	1.484	1.737	1.884	1.99	1.868	-

60	t	0	0.25	0.5	0.75	1	1.25	1.5	2	2.3	3	3.5	4	5	6	8	10	15	20	-
10.92	[Ag]	0	0.007	0.034	0.07	0.116	0.185	0.245	0.406	0.516	0.677	0.771	0.875	1.045	1.275	1.552	1.732	1.875	1.829	-

50	t	0	0.25	0.5	0.75	1	1.5	2	2.5	3	4	5	6	8	10	15	20	25	30	-
11.31	[Ag]	0	0	0.001	0.028	0.049	0.073	0.112	0.167	0.234	0.328	0.443	0.555	0.72	0.879	1.122	1.252	1.321	1.295	-

45	t	0	0.5	1	1.5	2	2.5	3	4	5	6	8	10	15	20	25	30	40	-	-
11.57	[Ag]	0	0.015	0.026	0.052	0.066	0.13	0.197	0.3	0.384	0.474	0.686	0.862	1.308	1.567	1.728	1.83	1.817	-	-

40	t	0	0.5	1	1.5	2	3	4	6	8	10	15	20	25	30	40	50	-	-	-
11.83	[Ag]	0	0.021	0.029	0.047	0.077	0.117	0.16	0.281	0.437	0.585	0.887	1.171	1.299	1.413	1.456	1.454	-	-	-

35	t	0	1	2	4	6	10	15	20	25	30	40	50	60	-	-	-	-	-	-
11.66	[Ag]	0	0.009	0.023	0.053	0.138	0.354	0.611	0.792	0.94	1.014	1.082	1.108	1.108	-	-	-	-	-	-

30	t	0	1	2	4	6	8	10	15	20	25	30	40	50	60	80	100	-	-	-
11.95	[Ag]	0	0.01	0.012	0.013	0.015	0.055	0.193	0.329	0.537	0.653	0.762	0.88	0.993	0.963	0.883	0.916	-	-	-

**Table 3:** Datasets of [Ag] vs. time for silver leaching evaluation in alkaline medium from sodium-silver jarosite using CN^−^ as complexing agent. [Ag] in mg L^−1^ and t in min.

[NaCN] (mol L^−1^) /pH_i_																					
0.1	t	0	1	2	4	6	8	10	15	20	25	30	40	50	60	70	80	100	-	-	-
11.27	[Ag]	0	0.022	0.034	0.061	0.076	0.166	0.408	1.154	2.196	3.387	4.483	6.28	7.307	7.948	8.228	8.488	8.612	-	-	-

0.04087	t	0	0.5	1	2	4	6	8	10	15	20	25	30	40	50	60	80	100	120	140	233
11.19	[Ag]	0	0.026	0.034	0.056	0.086	0.099	0.122	0.135	0.392	0.922	1.376	2.085	3.101	4.146	4.896	5.973	6.579	6.718	6.858	7.155

0.0286	t	0	2	4	8	12	16	20	25	30	35	40	45	50	60	70	80	100	120	140	160
11.19	[Ag]	0	0.007	0.013	0.021	0.029	0.093	0.26	0.575	1.138	1.825	2.831	3.595	4.739	5.81	6.691	7.442	8.314	8.775	9.07	9.106

0.01224	t	0	3	6	9	15	20	25	30	40	50	60	80	100	120	150	180	-	-	-	-
11.13	[Ag]	0	0.015	0.021	0.035	0.153	0.271	0.417	0.623	1.155	2.847	4.099	6.15	7.555	8.598	9.138	9.368	-	-	-	-

0.00408	t	0	5	10	15	20	25	30	40	50	60	70	80	100	130	160	200	240	300	-	-
11.13	[Ag]	0	0.007	0.019	0.02	0.03	0.065	0.091	0.31	0.609	1.074	2.215	2.875	4.226	6.194	7.791	8.836	9.28	9.505	-	-

0.00254	t	0	5	10	20	30	40	60	80	100	130	160	200	240	300	420	-	-	-	-	-
11.09	[Ag]	0	0.02	0.033	0.036	0.054	0.077	0.201	0.462	1.476	3.293	4.774	6.37	7.384	8.402	8.665	-	-	-	-	-

0.000408	t	0	5	10	20	30	40	60	80	100	130	160	200	240	300	360	420	480	-	-	-
11.09	[Ag]	0	0.005	0.006	0.009	0.018	0.078	0.185	0.417	0.583	2.059	3.509	5.082	6.175	7.061	7.655	7.904	8.007	-	-	-

[NaOH] (mol L^−1^) /pH_i_																					
0.03	t	0	0.5	1	1.5	2	3	4	6	8	10	15	20	25	30	40	50	70	-	-	-
12.50	[Ag]	0	0.089	0.129	0.238	0.571	1.141	1.705	3.131	4.65	6.025	7.548	8.076	7.674	7.747	7.747	7.498	7.687	-	-	-

0.01	t	0	0.5	1	1.5	2	4	6	8	10	12	15	20	25	30	40	50	70	110	-	-
11.99	[Ag]	0	0.013	0.028	0.046	0.093	0.192	0.392	0.71	1.241	2.373	3.515	4.659	5.792	6.354	6.737	6.631	6.756	6.666	-	-

0.0066	t	0	0.5	1	1.5	2	3	4	6	8	10	15	20	25	30	40	50	60	80	-	-
11.83	[Ag]	0	0	0.005	0.005	0.026	0.015	0.022	0.044	0.095	0.363	1.335	2.388	3.48	4.555	5.453	5.841	6.048	6.181	-	-
0.0011	t	0	0.5	1	2	4	6	8	10	15	20	25	30	40	50	60	80	100	120	140	233
11.19	[Ag]	0	0.026	0.034	0.056	0.086	0.099	0.122	0.135	0.392	0.922	1.376	2.085	3.101	4.146	4.896	5.973	6.579	6.718	6.858	7.155

0.00055	t	0	5	10	15	20	30	40	60	80	100	120	150	200	-	-	-	-	-	-	-
11.07	[Ag]	0	0.012	0.021	0.12	0.306	1.019	1.953	3.729	4.948	5.711	6.113	6.516	6.571	-	-	-	-	-	-	-

0.000275	t	0	5	10	20	30	40	50	60	80	100	120	150	200	240	-	-	-	-	-	-
11.00	[Ag]	0	0.015	0.042	0.202	0.502	1.118	1.879	2.869	4.095	4.913	5.38	5.811	6.054	6.119	-	-	-	-	-	-

0.00006	t	0	5	10	15	20	30	40	50	60	80	100	120	150	180	240	300	-	-	-	-
10.93	[Ag]	0	0.01	0.024	0.033	0.07	0.285	0.715	1.182	1.881	3.295	4.462	5.172	5.995	6.575	6.814	6.927	-	-	-	-

T (°C) /pH_i_																					
70	t	0	0.25	0.5	0.75	1	1.25	1.5	2	2.5	3	3.5	4	5	6	8	10	15	20	-	-
10.33	[Ag]	0	0.099	0.214	0.411	0.668	1.013	1.414	2.074	2.791	3.734	4.443	5.13	6.144	7.097	8.327	9.408	9.966	9.846	-	-

60	t	0	0.25	0.5	0.75	1	1.25	1.5	2	2.5	3	4	6	8	10	15	20	25	30	-	-
10.46	[Ag]	0	0.024	0.137	0.24	0.324	0.458	0.528	1.255	1.605	2.081	3.519	5.719	7.609	9.296	11.114	11.744	11.494	11.614	-	-

50	t	0	0.25	0.5	0.75	1	1.5	2	3	4	6	8	10	15	20	30	40	-	-	-	-
10.65	[Ag]	0	0.003	0.01	0.015	0.025	0.047	0.066	0.15	0.297	1.154	2.033	2.973	4.499	5.453	5.834	5.508	-	-	-	-

45	t	0	0.5	1	2	3	4	5	6	8	10	12	15	20	25	30	40	60	-	-	-
10.80	[Ag]	0	0.007	0.005	0.005	0.051	0.114	0.181	0.323	0.723	1.374	1.788	2.588	3.731	4.596	5.148	5.604	5.577	-	-	-
40	t	0	1	2	3	4	5	6	8	10	12	15	20	25	30	40	50	60	80	-	-
10.91	[Ag]	0	0.007	0.016	0.027	0.036	0.062	0.111	0.228	0.398	0.765	1.353	2.435	3.393	4.014	4.854	5.331	5.499	5.572	-	-

35	t	0	1	2	4	6	8	10	15	20	25	30	40	50	60	80	100	140	-	-	-
11.04	[Ag]	0	0.013	0.025	0.047	0.071	0.145	0.179	0.531	1.315	1.883	2.506	3.639	4.575	5.108	5.745	5.911	6.025	-	-	-

30	t	0	0.5	1	2	4	6	8	10	15	20	25	30	40	50	60	80	100	120	140	233
11.27	[Ag]	0	0.026	0.034	0.056	0.086	0.099	0.122	0.135	0.392	0.922	1.376	2.085	3.101	4.146	4.896	5.973	6.579	6.718	6.858	7.155

## 2 Experimental Design, Materials and Methods

### 2.1 Standards and reagents

Sodium cyanide, sodium thiocyanate, and sodium thiosulfate were used for the silver (Ag) leaching experiments; sodium hydroxide was used to adjust pH. All reagents were acquired from Sigma-Aldrich in ACS grade. For monitoring of Ag leaching by atomic absorption spectrometry (AAS), PerkinElmer Pure brand standard of Ag was used. All experiments were conducted using ultra-pure water with a resistivity of 18 MΩ cm.

### 2.2 Sample of synthetic sodium-silver jarosite

The sodium-silver jarosite sample was previously synthetized and fully characterized in a previous work [1]; details for the synthesis can be found in references [2,3]. The solid sample was sieved using Tyler sieves in the range −325/+400 mesh for the leaching experiments. The result of the sample elemental composition analysis was: Na 4.59%, Ag 0.89%, Fe 32.3 %, SO_4_^2−^ 40.4% and H + O 21.82% (obtained by difference). The chemical approximate formula corresponds to Na_0.96_Ag_0.04_Fe_2.82_(SO_4_)_2.01_(OH)_5.36_(H_2_O)_0.86_.

### 2.3 Silver leaching experiments

The evaluation of the silver leaching from the sodium-silver jarosite was performed following an experimental method employed in previous works related to the alkaline decomposition of jarosite type compounds, silver leaching from mining tailings and silver metallic studies with some modifications [4], [5], [6].

All silver leaching experiments were carried out in a Pyrex glass reactor of 500 mL placed over a heating plate with an automatic external probe of temperature (Thermo Fisher Scientific, model SuperNuova+), and coupled to a digital mechanical stirrer with a three-blade propeller (IKA model RW20). The pH of the leaching solution was constantly measured with a pH-meter Orion 3 Star equipped with an Orion Ultra Sure Flow electrode and an automatic temperature compensation probe (Thermo Scientific). For the experiments, 500 mL of the leaching solution prepared according to the conditions of sodium hydroxide and the complexing agent (Table 1, Table 2, Table 3), were decanted in the reaction vessel, and adjusted to a desired temperature. The stirring rate employed was 750 rpm. When the required temperature was reached, a sample of the leaching solution was taken corresponding to the condition t = 0 (blank). To start the reaction, 0.2 g of sodium-silver jarosite with an initial particle size of 38 μm were added; at this point, the reaction time begins to be measured. The progress of the silver leaching was carried out taken samples at different times (t). These aqueous samples were previously filtered to remove solid residues and the [Ag] was determined by AAS. The initial pH (pH_i_) in all leaching experiments was kept constant adding small volumes of a concentrated NaOH solution (1.0 mol L^−1^). To evaluate the effect of NaOH concentration on the silver concentration, temperature and concentration of the leaching agent were kept constant at 30 °C (303 K) and 0.1 mol L^−1^, respectively, varying the concentration of NaOH accordingly. To evaluate the effect of the leaching agent on the silver concentration in the leaching solution, the concentration of the lixiviant agent was varied maintaining constant the NaOH concentration and temperature (0.01 mol L^−1^, 30 °C). To evaluate the effect of temperature, the concentrations of NaOH and complexing agent were kept constant (0.01 and 0.1 mol L^−1^ respectively), varying the reaction temperature in each experiment.

## CRediT authorship contribution statement

**Hernán Islas:** Investigation, Conceptualization, Validation. **Mizraim U. Flores:** Investigation, Validation. **Julio C. Juárez:** Investigation. **Martín Reyes:** Investigation. **Alien Blanco:** Investigation. **Emmanuel J. Gutiérrez:** Writing – review & editing. **Javier Aguilar:** Data curation. **Mary C. Nolasco:** Investigation. **Israel Rodríguez:** Data curation. **Iván A. Reyes:** Writing – original draft, Supervision.

## Declaration of Competing Interest

The authors declare that they have no known competing financial interests or personal relationships which have, or could be perceived to have, influenced the work reported in this article.

## Data Availability

Dataset of Ag concentration against time (Original data) (Mendeley Data).
